# Analysis of Tillage Depth and Gear Selection for Mechanical Load and Fuel Efficiency of an Agricultural Tractor Using an Agricultural Field Measuring System

**DOI:** 10.3390/s20092450

**Published:** 2020-04-26

**Authors:** Yeon-Soo Kim, Wan-Soo Kim, Seung-Yun Baek, Seung-Min Baek, Young-Joo Kim, Sang-Dae Lee, Yong-Joo Kim

**Affiliations:** 1 Department of Biosystems Machinery Engineering, Chungnam National University, Daejeon 34134, Korea; kimtech612@gmail.com (Y.-S.K.); kelpie0037@gmail.com (S.-Y.B.); bsm1104@naver.com (S.-M.B.); 2 Convergence Agricultural Machinery Group, Korea Institute of Industrial Technology (KITECH), Gimje 54325, Korea; ojoo@kitech.re.kr (Y.-J.K.); sdlee96@kitech.re.kr (S.-D.L.); 3 Department of Smart Agricultural Systems, Chungnam National University, Daejeon 34134, Korea

**Keywords:** agricultural tractor, agricultural field measuring system, mechanical load, fuel efficiency, tillage depth, gear selection, travel speed, slip ratio, plow tillage

## Abstract

This study was conducted to analyze the effects of tillage depth and gear selection on the mechanical load and fuel efficiency of an agricultural tractor during plow tillage. In order to analyze these effects, we developed an agricultural field measuring system consisting of a load measurement part (wheel torque meter, proximity sensor, and real-time kinematic (RTK) global positioning system (GPS)) and a tillage depth measurement part (linear potentiometer and inclinometer). Field tests were carried out using moldboard plows with a maximum tillage depth of 20 cm and three gear selections (M2H, M3L, and M3H) in a rice stubble paddy field for plow tillage. The average travel speed and slip ratio had the lowest M2H and the highest M3L. M3H had the highest theoretical speed, but the travel speed was 0.13 km/h lower than M3L due to the reduction in the axle rotational speed at deep tillage depth. Regarding engine load, the higher the gear, the greater the torque and the lower the axle rotation speed. The front axle load was not significantly affected by the tillage depth as compared to other mechanical parts, except for the M3H gear. The rear axle load generated about twice the torque of the front wheel and overall, it tended to show a higher average rear axle torque at higher gear selection. The rear axle load and fuel rate were found to be most affected by the combination of the tillage depth and gear selection combination. Overall, field test results show that the M3H had the highest fuel efficiency and a high working speed while overcoming high loads at the same tillage depth. In conclusion, M3H is the most suitable gear stage for plow cultivation, and the higher the gear stage and the deeper the tillage depth during plowing, the higher the fuel efficiency. The results of this study will be useful for analyzing mechanical load and fuel efficiency during farm operations. In a future study, we will conduct load analysis studies in other farming operations that consider various soil mechanics factors as well as tillage depths and gear selections.

## 1. Introduction

Tractor work is usually performed with very high load fluctuations. The mechanical load and fuel consumption of the tractor are influenced by the operation type, travel speed, engine speed, opening degree of the throttle, tire pressure, and ballast [1]. The mechanical load during tillage operation is an indicator of overall working performance and a key factor in designing the power transmission of an agricultural tractor [2,3,4]. In general, the measurement of mechanical load measurement from field experiments is considered to be an important process for the optimal power transmission design of an agricultural tractor that could be used for a specific implementation [5]. Thus, it is essential into measure a mechanical load such that it reflects the actual operation conditions, such as the type of agricultural operation, travel speed, and so on. Among the various tractor operations, deep tillage requires the most energy and power in agricultural fields [6]. The parameters between the soil and the machine affect the mechanical load and fuel efficiency of the agricultural tractor [7].

Research through field measuring is essential in agriculture because agricultural machinery has different working and driving conditions depending on the attached implement, crop types, and local soil conditions. Various studies have been conducted through field measuring in the agricultural machinery field, including fault diagnosis, monitoring of working performance, and the development of decision-support system for agricultural machinery [8,9,10,11]. In another study, a field measuring of hydraulic pressure during was conducted to diagnose failures in the transmission system of tractor [12]; the results showed an 88% reduction of repair time and a 93% reduction of repair cost. In another study, a decision-support system was developed and applied to assist producers with expected yield improvements using a global positioning system [13]; a suitable agricultural machinery matching with the working area through an application of the development system, it was found that the carbon emission was reduced to improve the working environment, and fuel efficiency was increased to reduce operating costs. In addition, many studies have been conducted to perform field experiments using field measuring systems in studies related to agricultural tractors, which is the most expensive and widely used agricultural machinery. In order to optimize design and decision support for fault diagnosis of agricultural tractors, studies on the measurement of mechanical loads of major parts according to the type of tillage operations were also carried out using field measuring systems. The following studies have been conducted with regard to the mechanical loads of agricultural machinery during farm operations. A study on the automatic fault diagnosis of the hydraulic system of a tractor was conducted using a multiple signal classification algorithm and pseudo-spectrum analysis method [14,15]; the results show that the oil filter sound analyzer application provides an economical approach to predicting and detecting the choked stage of agricultural machinery hydraulic systems. A study was conducted to analyze the loads that act on the transmission and driving axle of an agricultural tractor during plowing tillage [16]; the results revealed that the effect of travel speed on the load spectrum was more significant for the input shaft of the transmission than for the final drive shaft. A field experiment was conducted to study the effects of a mechanical load resulting from the power take-off (PTO) gear selection and operation type on the fatigue life of the PTO driving axle during rotary tillage [17,18]. The results also showed that the PTO driving axle experienced a more severe load in paddy fields than in upland sites, and there was a greater relative severity at higher PTO rotational speeds. Another study analyzed the PTO severity of an agricultural tractor during rotary tillage and baler operation [19]; the results showed that the damage to the PTO increased when the transmission or PTO gear increased. The severity of the PTO during rotary tillage was shown to be greater than that of the baler operation. The load of the PTO shaft for a riding type transplanter was analyzed according to the planting distance [20,21]; the results showed that a shorter planting distance had a more significant effect on the PTO shaft than a longer planting distance. In another study, a strength analysis of the mechanical transmission of an 82 kW class agricultural tractor was conducted using a measured equivalent torque during plow tillage [22]; the results showed that the design using the maximum torque of the engine was much stronger than the design using the measured load data in real agriculture, indicating that an optimal design is needed. In another study, a field experiment was conducted to measure the load of a PTO input shaft on a multipurpose cultivator [23]; the measured mechanical load was used to evaluate the strength of PTO gear trains based on transmission and PTO gear combinations. While studies have been conducted on the effects of factors such as gear selection, operation type, and planning resistance on a mechanical load for agricultural machinery, tractor field tests considering tillage depth, one of the major factors that has a significant impact on crop productivity, have not recently been performed [24]. Several studies on the mechanical load of agricultural machinery have considered tillage depth and travel speed. The effect of tillage depth and travel speed on draft force was analyzed during tandem disc, chisel plow, and field cultivator operations [25]; the results showed that tillage depth and travel speed had a great influence on draft force, but there were limitations when directly measuring tillage depth, and only the influence on draft force was analyzed with the exclusion of the mechanical load of major components. The energy requirements of an agricultural tractor were analyzed considering the crop, tillage timing, soil compaction, and tillage depth during cotton yield operation [26]. In other studies, soil bin investigations were conducted to measure draft force according to operation conditions such as speed, penetration angle, soil water content, cone index, and tillage depth [27,28,29].

Various studies have been conducted on tractor efficiency as well as on tractor operating strategies such as gear up and throttle down [30,31]. A simulation study was performed to minimize the fuel consumption of tractors through the application of an automatic gear shift strategy [32]; according to the simulation result analysis, the fuel consumption decreased and the working speed increased, but the field load and the working environment were not verified through the field test. In some studies, the effect of the controlled traffic system on fuel saving was studied [33,34]; the controlled traffic system showed a fuel saving of about 19.79 L/ha (23.7%) in field experiments with zero tillage. A study analyzing fuel consumption according to the conditions of harrow disc angle, tillage depth, and travel speed, was performed when working with disc harrow [35]; the results showed that the optimum working conditions with the lowest fuel consumption were found at a 10° disc angle, 3.5 m/s travel speed, and 8 cm tillage depth. In another study, a study was conducted on fuel consumption reduction through the development of a tire inflation pressure model [36]; the results confirm that under specific tire pressure conditions, the fuel consumption could be reduced by 2.5%–3.0% per hour and by 3.0%–4.0% per hectare. A study was carried out to develop a Hall sensor embedded system to measure the wheel slip for reducing the fuel rate [37]; the results of the soil bin test for validation show that the amount of fuel saving was up to 1.3 L/h. Another study examined the effect of the gang angle on fuel consumption and work rate, during offset disc harrow [38]; the results of the field experiment showed that the faster the work rate and the lower the angle between the gangs, the lower the fuel consumption per hectare.

Various studies on mechanical load and fuel efficiency of agricultural machinery have been performed so far, but real-time precision tillage depth measurement is impossible; thus, only the effect on rough depth units in each soil layer unit has been considered. Several studies have considered the tillage depth condition, but the analysis was performed according to tillage depths that were simply measured directly or limited to the soil bin test. Studies carried out on indoor soil bins are limited as they did not consider the actual working speed and soil conditions, such as paddy field or upland field. In addition, most studies only conducted a draft force analysis and did not consider axle load, which is an important design consideration in the engineering of wheeled vehicles. In addition, although load data (as measured through field tests) are used to evaluate the reliability of dynamometer components, the measurement is generally carried out using the average load of the entire working section. In the proper gear when the actual working machine was being used, load analysis was not carried out according to the main factors such as tillage depth and gear selection, and it was difficult to determine whether the average load was also generated under the specific agricultural working conditions. Therefore, an updated study from field tests is needed to study the mechanical load and fuel efficiency while considering the effects from the combination of tillage depth and gear selection.

The purpose of this research is to analyze the mechanical load and fuel efficiency of an agricultural tractor with tillage depth and gear selection and develop operation strategies to improve fuel efficiency. The specific objectives of this study were to (1) develop a field measuring system for mechanical load according to tillage depth and gear selection; (2) measure mechanical load (engine load, fuel rate, and axle load), tillage depth, and travel speed with the slip ratio of an agricultural tractor during a field experiment; and (3) analyze the effects of tillage depth and gear selection on the mechanical load and fuel efficiency of an agricultural tractor during plow tillage.

## 2. Materials and Methods

### 2.1. Agricultural Tractor and Implement

A 78 kW class agricultural tractor (S07, Tong Yang Moolsan, Gongju, Korea) was used in this experiment. The tractor had an empty vehicle weight of 3890 kg, and the total weight including all attached measuring instruments was 5260 kg, and the dimensions were 4225 mm × 2140 mm × 2830 mm (length × width × height). The agricultural tractor used for the field experiment was equipped with a mechanical transmission of power shuttle and power shift type. A total of 64 travel speeds (32 forward, 32 backward) of the tractor were determined by the combination of gears which were selected depending on the farm operation. The specifications of the agricultural tractor used in this experiment are shown in Table 1.

The attached implement used for plow tillage during the field experiment was a moldboard plow. Moldboard plows are mainly used in rice stubble paddy fields, as they are superior to other plow implements in terms of work stability. However, high mechanical loads occur during tillage operations due to high soil resistance. An 8-row moldboard plow (WJSP–8, Woongjin Machinery, Gimje, Korea), which is suitable for a 78 kW agricultural tractor was used in this experiment. The specifications of the moldboard plow are shown in Table 2.

**Figure 1:**
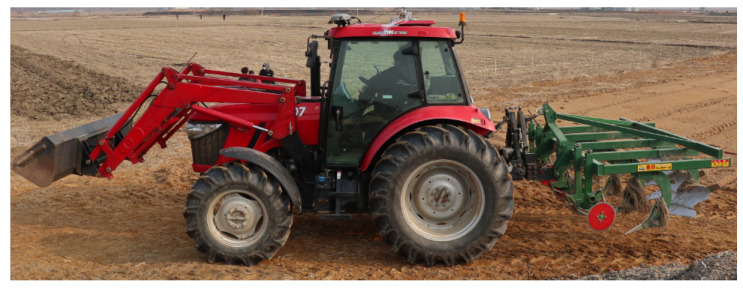
The 78 kW class agricultural tractor used for the field experiment.

The agricultural field measuring system, configured for the study on the effect of tillage depth and gear selection on mechanical load, consisted of two main parts: the tillage depth measurement part, which measures the soil penetration depth of the attached implement, and the load measurement part, which measures the mechanical load, fuel rate, and travel speed. A 78 kW class agricultural tractor with a field measuring system used for plow tillage is shown in Figure 1.

### 2.2. Measurement of Soil Properties

**Figure 2:**
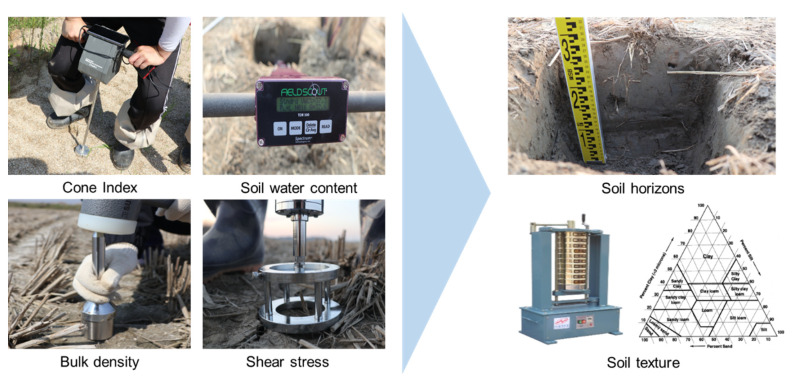
Overall test procedure of the soil property measurement.

One of the most relevant causes of soil compaction in agriculture is the repeated use of heavy machinery, which increases the pressure on the soil layer, not only for the top soil but also the subsoil [39]. Increased soil compaction can lead to increased hardpan in deep soil layers, which can lead to various problems such as soil porosity, poor drainage, and reduced yields due to lower crop root growth [40,41,42,43,44,45,46]. Soil properties such as cone index, bulk density, soil shear stress, soil water content, soil horizons and soil texture are the main soil mechanics factors that affect the interaction between soil and agricultural machinery, so the process of measuring the physical properties of soil is very important [47]. The overall test procedure for soil measurements, in analyzing soil properties, is shown in Figure 2. The cone index is the soil strength index (kPa), which is the force required to press the soil with a 30° circular stainless steel cone with a driving shaft [48,49], and is measured by using cone penetrometer (DIK 5532, Daiki Rika Kogyo Co., Ltd., Saitama, Japan). A soil water content measurement was performed in paddy soil where plow tillage was performed; this was measured by a soil water sensor (FieldScout TDR 350, Spectrum Technologies, Aurora, IL, USA). In addition, bulk density and shear stress, which are soil properties that have a great influence on soil strength, were measured by using a stainless sampling tube (DIK-1801, Daiki Rika Kogyo Co., Ltd., Saitama, Japan), a supplementary soil sampler (DIK-1630, Daiki Rika Kogyo Co., Ltd., Saitama, Japan), and digital soil resistance meter (DIK-5503, Daiki Rika Kogyo Co., Ltd., Saitama, Japan). Bulk density, which is an indicator of soil compaction, and shear stress in soil—a term used in soil mechanics to describe the magnitude of the shear stress that a soil can sustain—were obtained from Equations (1) and (2) [50,51].

(1) $$\mathrm{BD}\;={\;W}_{\mathrm{soil}}/V_{\mathrm{soil}}$$

where BD is the bulk density ($\mathrm{kg}/m^{3}$); $W_{\mathrm{soil}}$ is the sampled soil weight, including soil water and organic matter (kg); and $V_{\mathrm{soil}}$ is the volume of soil in the sampling stainless tube ($m^{3}$).

(2) $$T_{\mathrm{soil}}=\frac{3T}{2\pi \left(r_{o}{}^{3}-r_{i}{}^{3}\right)}$$

where $T_{\mathrm{soil}}$ is the shear stress of soil (kPa), T is the shear stress that occurred during vane shear test (Nm), $r_{o}$ is the radius of the outer diameter of circular shear ring (m), and r_i_ is the radius of the inner diameter of the circular shear ring (m).

The sampled soil was analyzed for soil texture using a sieve shaker (HJ-2152, Heungjin, Gimpo, Korea). In the laboratory test conducted after the field test, the soil horizons were analyzed for the depth of the hardpan by analyzing the measured cone index and bulk density. In addition, soil texture analysis was conducted using the US Department of Agriculture (USDA) standard [52].

### 2.3. Agricultural Field Measuring System

In this study, an agricultural field measuring system composed of a tillage depth measurement part and a load measurement part was attached to the tractor to analyze the effect of tillage depth on the mechanical load during tillage operation.

#### 2.3.1. Tillage Depth Measurement Part

**Figure 3:**
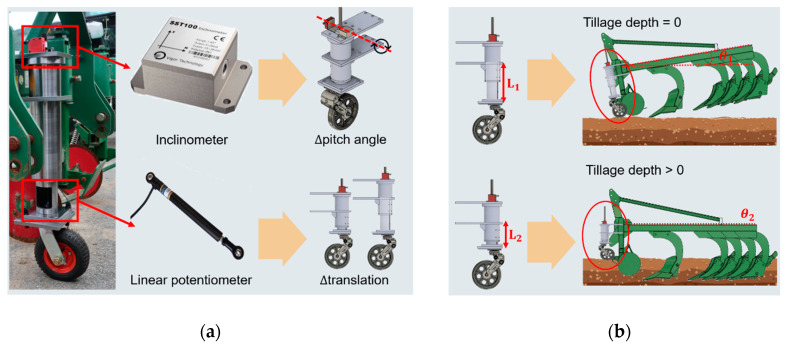
Tillage depth measurement part of the agricultural field measuring system: (**a**) configuration of measurement system and (**b**) principle during tillage operation.

The detailed configuration of the tillage depth measurement part of the field measuring system is shown in Figure 3 [53]. The main principle of the tillage depth measurement part is to calculate the relative vertical displacement difference of the share point of the first shank. The tillage depth is calculated in real-time using the pitch angle of the attached machine and the vertical penetration depth. The sensors used in the tillage depth measurement part are as follows. The sensors used in the tillage depth measurement part are as follows.

- Inclinometer

The pitch angle of the attached implement was measured by using a 1 axis inclinometer (SST141, Vigor Technology, Shanghai, China), which has measurement range from 0°–180° and an accuracy of ±0.05° at 25 °C. It has direct analog voltage outputs (0.5–4.5 VDC) that are proportional to the pitch angle of the implement.

- Linear potentiometer

The vertical penetration depth of the attached implement was measured by a linear potentiometer (CLS1322, Active Sensors, Christchurch, UK), which has a measuring range of 25–350 mm and an accuracy of <±0.05%. In addition, it can operate up to 125 °C with a durability rating of IP65, so it is suitable for harsh test environments. Equation (3) was used to measure the tillage depth using the tillage depth measurement part in this experiment [53]:

(3) $$\mathrm{TD}\;={\;L}_{1}\cos\theta_{1}-L_{2}\cos\theta_{2},$$

where TD is the tillage depth (mm); L_1_ is the displacement of the measurement system as measured by a linear potentiometer when the tillage depth is zero (mm); θ_1_ is the pitch angle of the tillage depth measurement part when the tillage depth is 0, which can be measured by an inclinometer (degrees); L_2_ is the displacement of the measurement system as measured by a linear potentiometer (mm); and θ_2_ is the pitch angle of the measurement system as measured by an inclinometer (degrees).

#### 2.3.2. Load Measurement Part

**Figure 4:**
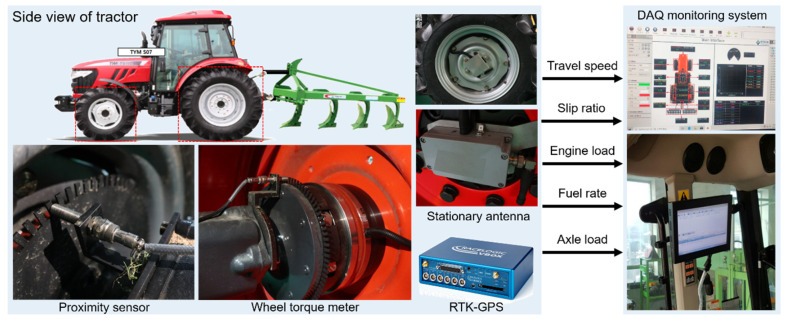
Configuration of load measurement part of the agricultural field measuring system.

The engine and the axle load are important design considerations in the engineering of a wheeled vehicle. The load analysis process in a field experiment is important not only when analyzing the load input, but also in the simulation process, performance evaluation, and durability assessment in vehicle engineering [54]. The load measurement part was configured to essentially measure engine load, fuel rate, axle load, and even the travel speed and slip ratio of an agricultural tractor. The sensors used in the load measurement part are as follows. The detailed configuration of the load measurement part of the agricultural field measuring system is shown in Figure 4.

The load measurement part of the engine was configured to measure the engine load (engine torque and engine rotation speed) and fuel rate by communicating with an electronic control unit based on wireless controller area network (CAN) control communication. The measured engine load and fuel rate were used to determine specific fuel consumption (SFC), which is mainly used when analyzing actual tractor fuel efficiency [55,56]. Although engine efficiency affects fuel consumption, fuel consumption alone is not a good indicator of fuel efficiency. Therefore, specific fuel consumption was developed as a fuel efficiency index that consider the amount of work that is being done by the engine. The specific fuel consumption of the agricultural tractor was obtained using the following [57]:

(4) $$\mathrm{SFC}\;=\frac{\mathrm{FC}}{{\mathrm{PWR}}_{\mathrm{engine}}},$$

where SFC is the specific fuel consumption (g/kWh), FC is the fuel rate (L/h), and ${\mathrm{PWR}}_{\mathrm{engine}}$ is the engine power (kW).

(5) $${\mathrm{PWR}}_{\mathrm{engine}}=\frac{2{\pi T}_{\mathrm{engine}}N_{\mathrm{engine}}}{60000},$$

where $T_{\mathrm{engine}}\;\mathrm{is}\;\mathrm{the}\;\mathrm{engine}\;\mathrm{torque}$ (Nm) and $N_{\mathrm{engine}}$ is the engine rotational speed (rpm).

- Wheel torque meter

The wheel torque meter (PCM16, MANNER, Spaichingen, Germany) used to measure a wheel torque. The strain gauge type torque meter for measuring wheel axle torque has a measurement range from 500 Nm to 4 kNm and is an output with an analog voltage (0 to ±10 V).

- Proximity sensor

The proximity sensor (PRDCML30–25DN, Autonics, Busan, Korea) used to collect the rotational speed of the wheel axle has a sensing distance of 25 mm and a response frequency of 100 Hz. The maximum axle rotation speed that could be measured was 4000 rpm, and the operating temperature was in the range of −25 to 70 °C.

- Real-time kinematic global positioning system (RTK-GPS)

The slip ratio is one of the main indicators in evaluating the performance of an agricultural tractor during tillage operation [58]. In order to ensure sufficient and constant traction performance, the tractor must be able to reach a high enough travel speed. The theoretical speed of the three-gear selection is different, but the agricultural tractor’s performance varied greatly depending on the slip ratio generated on an irregular road surface. Thus, the slip ratio was important in determining the travel speed of the agricultural tractor during farming operation [59]. Accurate theoretical speed and actual travel speed were required to obtain the slip ratio. The theoretical travel speed used in the slip ratio calculation was obtained using Equation (6) [60]. A global positioning system (GPS) sensor (VBOX 3i, VBOX Automotive, Buckingham, UK) was used to measure the actual travel speed required to obtain the slip ratio from Equation (7) [61]. The VBOX 3i uses a GPS/Global Navigation Satellite System (GNSS) receiver, logging data at 100 times a second, and has an accuracy of 0.1 km/h error (averaged over 4 samples). It also has a minimum measurement travel speed of 0.1 km/h, making it suitable for measurement testing related to agricultural machinery at speeds less than 10 km/h for most operations. The theoretical speed of the agricultural tractor was obtained using the following equation:

(6) $$V_{\mathrm{Theoretical}}={\;\pi \mathrm{DN}}_{\mathrm{wheel}}=\frac{{\pi \mathrm{DN}}_{\mathrm{engine}}\mathrm{GR}3.6}{60},$$

where V_tire_ is the theoretical speed of the agricultural tractor (km/h), D is the diameter of the wheel (m), N_wheel_ is the rotational speed of the axle as obtained from proximity sensors (rpm), and GR is the gear ratio at the selected gear.

The slip ratio of the agricultural tractor was obtained using the following equation:

(7) $$S\;=\left(1-\frac{V_{\mathrm{vehicle}}}{V_{\mathrm{tire}}}\right)100,$$

where S is the slip ratio of the agricultural tractor during tillage operation (%), $V_{\mathrm{vehicle}}$ is the travel speed of the tractor obtained from the real-time kinematic (RTK) GPS sensor (km/h), and V_tire_ is the theoretical speed of the tractor obtained from Equation (6) (km/h).

### 2.4. Test Procedure

To measure mechanical load and fuel efficiency according to tillage depth and gear selection, field experiments were conducted in Kumam-ri, Songsan-myeon, Dangjin-si, Chungcheongnam-do, Korea. The size of the test site was 100 m × 80 m, and its latitudinal and longitudinal coordinates were 36°55′48″ N and 126°37′59″ E, respectively. First, we analyzed how the main soil properties affecting soil–machine interaction differed according to soil depth. The target tillage depth was determined according to the results, and a field test was performed. Plow tillage was performed in four wheel drive mode and using three gears (M2H, M3L, and M3H) with theoretical speeds of 6.01, 7.09, and 8.43 km/h on a straight path that was 100 m long. In addition, field tests were performed under full-throttle conditions, commonly used by tractor drivers. The target tillage depth during the field test was set deeper than that estimated by the top hardpan from the cone penetration test. Although excessive load or slip occurs when the tillage depth is too deep, the moldboard implement used in this study was performed at a maximum descent point of a three-point hitch because it was equipped with skids to prevent more than 20 cm penetration. The target analysis section of the tillage depth is the hardpan layer, which is a surface layer of a paddy field that is physically affected by the use of agricultural machinery annually or periodically through farm operations [62,63]. In detail, the target analysis section of the tillage depth was selected and corresponded to the hardpan, which is the depth at which the mean cone index value increases instantaneously [64]. The effects of tillage depth and gear selection on the mechanical load of the agricultural tractor were analyzed at the depth estimated to be the hardpan layer. To analyze these effects, the measured data were arranged according to tillage depth. In addition, statistical methods using descriptive statistics with ANOVA using Duncan’s multiple range test and linear correlation with Pearson’s correlation coefficient were performed to analyze the effects of independent variables (tillage depth, gear selection) on dependent variables (engine load, fuel rate, axle load, travel speed, and slip ratio) through IBM SPSS Statistics software (SPSS 25, IBM Corp., Armonk, NY, USA). Basically, the effect of tillage depth and gear selection on the mechanical load and fuel efficiency of the agricultural tractor were analyzed, according to which the three gears were used in the deep tillage section to overcome the largest load at the fastest speed in the same working environment. Based on these results, the most fuel-efficient gear was selected while overcoming the appropriate load during plowing.

## 3. Results

### 3.1. Soil Properties

**Figure 5:**
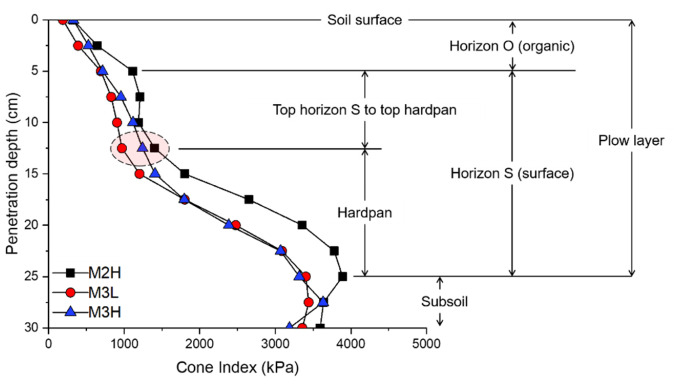
Analyzed soil profile and hardpan depth based on the cone index measurement results.

From the perspective of soil mechanics, the measurement results of the main soil property affecting the mechanical load were analyzed, as shown in Table 3, according to the soil depth of the four levels. Cone index results showed 558.12 kPa at 0–5 cm, and the average increase was 3.63 times that of the deepest: 15–20 cm to 2026.34 kPa. As a result of analyzing the soil profile, which is called the soil horizon [65] based on the cone index results, it was analyzed that the horizon O layer was about 0–5 cm and the hardpan estimated depth was about 12–13 cm to the maximum peak point of 25 cm as shown in Figure 5. Accordingly, the field test measurement data was analyzed for data from 13 cm that was estimated to be the depth of the hardpan layer. The bulk density at horizon O was 1650 $\mathrm{kg}/m^{3}$, and the bulk density at horizon S was similar at 1800–1830 $\mathrm{kg}/m^{3}$. However, the average bulk density increased to 1900 $\mathrm{kg}/m^{3}$ in the 15–20 cm section, which was near the subsoil at horizon S. The shear strength also increased from 43.87 to 77.99 kPa. The shear strength of each section was analyzed as a medium undrained shear strength level up to 0–15 cm, and a 15–20 cm high undrained shear strength level over 75 kPa [66]. Soil water content was about 17% higher than horizon O at horizon S, but there was no significant difference in the depth within horizon S. The results of soil texture analysis, using the USDA standard method, showed that the sampled soil was classified as loam (34% sand, 48% silt, and 18% clay).

### 3.2. Tillage Depth

**Figure 6:**
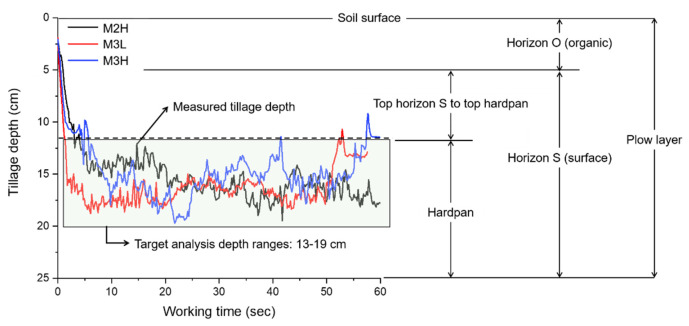
Results analysis of the measured tillage depth in each gear.

The average penetration resistance increased with increased tillage depths, but due to the irregular distribution of the actual hardpan and irregularity of the road surface, the measured tillage depth data show irregular shape. The field experiments performed under all gear conditions seem to have been performed in the plow layer. The measured tillage depth was 15.61 ± 1.51, 16.58 ± 1.47, and 15.44 ± 1.89 cm in the three gears. When shifting from M2H to M3L, the average tillage depth increased by 0.97 cm; however it tended to decrease by 1.14 cm when shifting from M3L to M3H. The descriptive statistics of measured tillage depth are shown in Table 4. Descriptive statistics results show that the standard deviation of the mean tillage depth was about 1–2 cm. Thus, the measured field test data were grouped by 1 cm to eliminate data irregularities in the analysis using the group frequency distribution method to clearly analyze the effect of tillage depth and gear selection on the elements related to mechanical load and fuel efficiency. In addition, the analysis depth range was set from 13 cm, which was determined to be the top hardpan depth, to 19 cm, the maximum tillage depth, for the data measured by the field experiment. Overall, the average tillage depth compared to the target center depth shows similar values, not only having constant hardness due to an irregular soil surface and instantaneous rapid load of a specific part, but also having various deviations from 13 to 19 cm. The target tillage depth is different for each operator or farming environment, so data trend analysis is required for precision measurements based on mechanical load and fuel efficiency. Therefore, it is necessary to conduct precise field test measurement-based mechanical load and fuel efficiency analysis studies rather than 5–10 cm rough test designs, e.g., such as simple shallow, medium, and deep. The specific results of the measured tillage depth are shown in Figure 6.

### 3.3. Travel Speed with Slip Ratio

**Figure 7:**
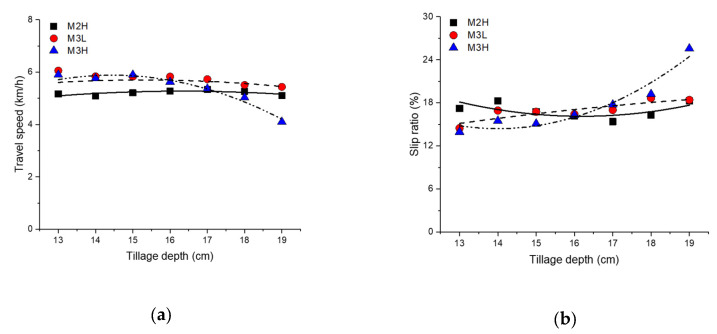
Mean values of (**a**) travel speed and (**b**) slip ratio according to tillage depth and gear selections during plow tillage.

The average travel speed and slip ratio according to tillage depth and gear selection are shown in Figure 7. Plow tillage in the M2H gear showed an average travel speed of 5.23 km/h and an average slip ratio of 16.71%. The overall average travel speed of M2H was 6.7%–8.8% lower than that of M3L and M3H. The mean travel speed of M2H in accordance with the tillage depth, did not change within 5%, indicating no significant trend in the tillage depth. In addition, since the slip ratio did not seem to change linearly with the tillage depth, the travel speed in M2H did not seem to have a significant effect considering soil resistance. As the tillage depth increased to 19 cm in case of M3L, the travel speed gradually decreased by up to 10.3% and the slip ratio gradually increased by about 27%. Plow tillage in M3H showed an average travel speed of 5.6 km/h and an average slip ratio of 16.54%. While the slip ratio of M3H is lower than that of M3L, the overall travel speed is low, suggesting that the theoretical speed in M3H significantly affects the traction and axle loads due to soil resistance. In particular, the average slip ratio in M3H rapidly increased by 25.57% in the deep tillage depth section (17–19 cm). It should be considered that relatively excessive slip occurs when the tillage depth is deeper than 17 cm in M3H. Summarized in Table 5 and Table 6 are the detailed average travel speed and slip ratio results according to tillage depth and gear selection with ANOVA analysis using Duncan’s multiple range test.

### 3.4. Engine Load and Fuel Rate

#### 3.4.1. Engine Load

**Figure 8:**
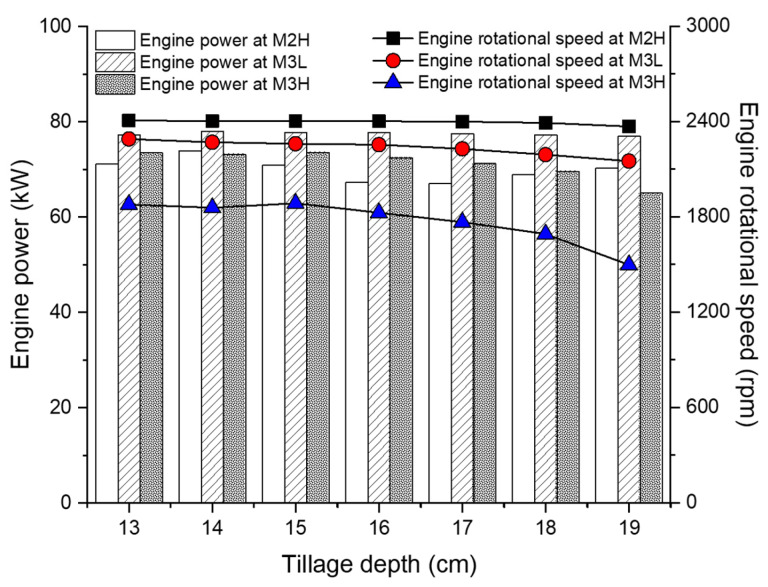
Overall engine load according to tillage depth and gear selection.

The average engine load was analyzed according to tillage depth and gear selection as shown in Figure 8. The overall average engine load at M2H was 227.46 Nm at 2401.86 rpm. The average engine torque at M2H did not show trends in the tillage depth. The difference between the minimum and maximum average engine torque was 27.01 Nm (9.2% difference). In addition, the difference in the average engine rotational speed between the minimum and maximum at M2H was 32.92 rpm (1.4% difference). Based on these results, the engine load at M2H is considered to have a relatively insignificant effect in terms of tillage depth and gear selection on mechanical load and fuel efficiency. For M3L, the overall average engine load was 331.86 Nm at 2233.06 rpm. The average engine torque at M3L tended to decrease linearly at increased tillage depths. As the tillage depth increased by 1 cm, the average engine torque increased by 3.21 Nm, or 0.96%, as compared to the overall average engine torque at M3L. As opposed to torque, the average rotational speed at M3L tended to increase linearly as the tillage depth increased. As the tillage depth increased by 1 cm, the average engine rotational speed decreased by 23.16 rpm, which is 1.03%, as compared to the overall average engine rotational speed at M3L. For M3H, the overall average engine load was 380.63 Nm at 1818.82 rpm. The average engine torque at M3H tended to increase more rapidly with the tillage depth than at M3L. As the tillage depth increased by 1 cm, the average engine torque at M3H increased by 7.09 Nm and the average engine rotational speed decreased to 64.73 rpm. The rate of increase of the average engine torque for the tillage depth at M3H was 2.11%, which was higher than at M3L (0.97%). The rate of decrease of the average engine rotational speed for the tillage depth at M3H was 0.95%, which was almost the same as at M3L (0.98%). However, the engine rotation speed of the M3H stage in the deep tillage depth section (17–19 cm) decreased by an average of 129.46 rpm as it was 1 cm deep, which is four times higher than M3L. Overall, the engine load was greatly affected by tillage depth and gear selection. As the gear stage increased, the overall average engine torque increased by 19%–37% as compared to the M2H, and the overall average engine speed decreased by 7.1%–24.3% as compared to M2H. Summarized in Table 7 and Table 8 are the detailed average engine load and engine power results according to tillage depth and gear selection with ANOVA analysis using Duncan’s multiple range test.

#### 3.4.2. Fuel Rate with Engine Power

The overall average engine power and fuel rate are shown in Table 9 and Table 10. The overall average engine power and fuel rate at M2H with the lowest engine load were 69.75 kW and 19.24 L/h, which corresponds to the lowest of the three gear stages. While the engine torque increased rapidly as the gear selection increased, the average engine power was 6.9% higher than M3H due to the decrease in the engine rotational speed. The fuel rate in M3L showed higher engine power and fuel rate than M3H in all tillage depth sections, even though the engine load was lower than M3H. In addition, although the M3H output was higher than the M2H, it showed the lowest fuel rate of the three gear stages. In all the gear stages, the overall tillage depth increased, and the fuel rate tended to decrease. It can be seen from Table 7 and Table 8 that the engine torque increases as the tillage depth increases, and the engine power also tends to decrease as the rpm decreases.

### 3.5. Axle Load

The axle load in actual operation is an important factor in the optimum design of agricultural tractors. In general, these tractors are designed with different sizes of front and rear wheels to disperse the pressure applied to the road surface by securing more contact area with higher loads due to the attached implement effect, and to ensure a smaller turning radius by making the front wheel smaller. In addition, the tractor center of gravity lies toward the rear axle. The rear tire size should be larger, which causes a difference in the load on the front and rear axles. Therefore, it is necessary to measure and analyze the axle load of the front and rear wheels separately, because the loads (torque and rotational speed of each wheel axle) that occur vary during farming operation.

#### 3.5.1. Front Axle Load

**Figure 9:**
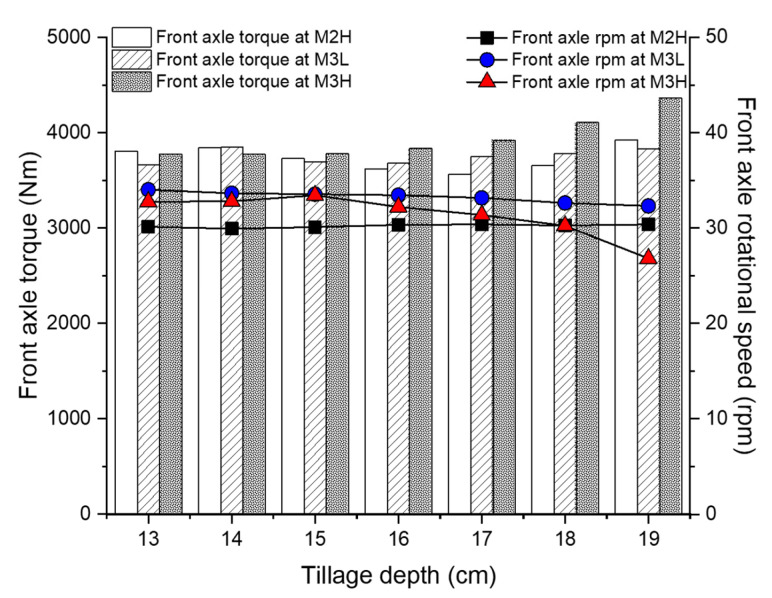
Overall front axle load according to the tillage depth and gear selections.

The average front axle load was analyzed according to the tillage depth and gear selection as shown in Figure 9. The overall average front axle load at M2H was 3697.7 Nm at 30.19 rpm. The mean front axle torque in M2H increased to 19.9 Nm as the tillage depth increased by 1 cm, but there was no tendency toward increased torque according to the tillage depth in the 13–17 cm range. The mean forward axle rotation speed in M2H was generally found to be constant regardless of the tilling depth, as the average depth decreased by only 0.03 rpm as the tillage depth increased by 1 cm (maximum 0.44 rpm). The overall average front axle load at M3L was 3728.1 Nm at 33.19 rpm. The average front axle torque of M2H increased to 24 Nm as the tillage depth increased by 1 cm in the target analysis section. The minimum–maximum difference was 8.4%, which was the smallest of the three gear selections. The average front axle rotational speed was less than 1% of the difference depending on the tillage depth. The overall average front axle load at M3L was 3861.2 Nm at 32.18 rpm. As the tillage depth increased by 1 cm, the average axle torque increased by 25.81 Nm and the average axle rotational speed decreased by 0.29 rpm. The front axle load at M2H and M3L did not show a significant difference according to the tillage depth. However, as the front axle load in M3H increased the tillage depth by 1 cm, the average torque increased by 107.8 Nm and the average rotation speed decreased by 1.19 rpm. The difference between the maximum and minimum torques of 589.9 Nm (13.53%) and rotational speed of 6.62 rpm (19.81%) seemed to be relatively more affected than for the other gear selections. Overall, the higher the gear, the greater the front axle load for the same tillage depth. From these results, the front axle load was found to vary slightly depending on the conditions of the tillage depth and gear selection. The detailed average front axle load results according to the tillage depth and gear selection, with ANOVA analysis using Duncan’s multiple range test, are summarized in Table 11 and Table 12.

#### 3.5.2. Rear Axle Load

**Figure 10:**
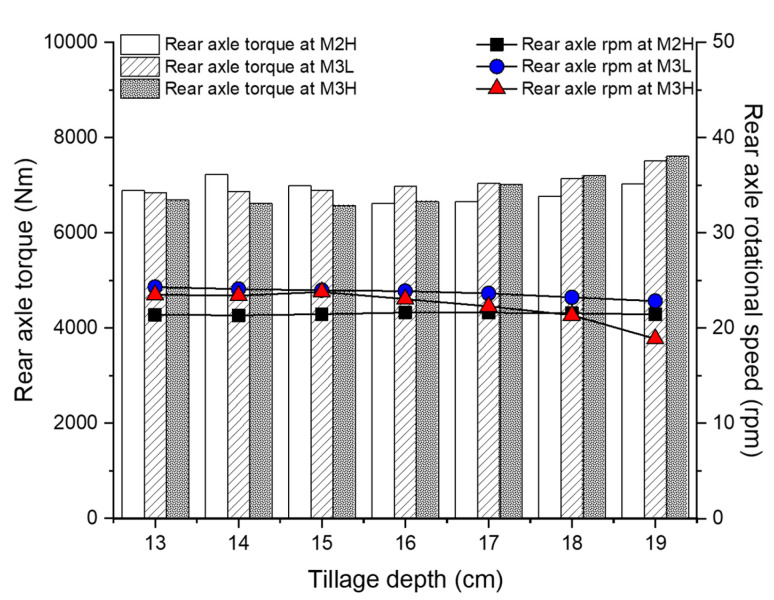
Overall rear axle load according to the tillage depth and gear selections.

The average rear axle load was analyzed according to the tillage depth and gear selection as shown in Figure 10. The overall average rear axle load at M2H was 6857.2 Nm at 21.51 rpm. The average rear axle torque of M2H was low at 24.21 Nm per 1 cm in all the analyzed sections, but increased to 271 Nm at 17–19 cm, indicating it was significantly affected in the deep tillage depth section. Although the average rear axle torque increased with the tillage depth like the other mechanical elements, the average rear axle rotational speed of M2H was hardly affected by the tillage depth, as compared to the other gear stages. In the case of M3L, the overall average rear axle load was 7025.76 Nm at 23.67 rpm. As the tillage depth increased by 1 cm, the average rear axle torque of M3L increased linearly by an average of 123.4 Nm (3.2%) and increased up to 664.42 Nm (9.7% compared to the minimum average torque). In addition, as the tillage depth increased by 1 cm, the average rear axle rotation speed of M3L decreased by an average of 0.25 rpm and decreased up to 1.46 (6.01% compared to maximum average rotational speed). The overall average rear axle load at M3H was 6754.8 Nm at 22.94 rpm. The rear axle torque at M3H tended to increase rapidly from a tillage depth of 16 cm. Overall, an increase of 1 cm in tillage depth increased the average 166.83 Nm, and the total increase was 1034.03 Nm (15.73%) compared to the lowest average torque. In addition, the average rear axle rotational speed at M3H decreased rapidly from 16 cm, as with torque, with an average decrease of 0.89 rpm for every 1 cm increase in the tillage depth, and the total decrease relative to the highest average rotational speed (23.8 rpm at 15 cm) was 4.93 rpm (20.72%). The average rear axle load was significantly more affected by tillage depth and gear selection than the average front axle load. In particular, the higher the gear selection, the deeper the tillage depth section, and the greater the effect of the tillage depth and gear selection on the mechanical load of the rear axle. The detailed average rear axle load results according to the tillage depth and gear selection with ANOVA analysis using Duncan’s multiple range test, are summarized in Table 13 and Table 14.

### 3.6. Statistical Analysis

The Pearson correlation coefficient was computed to assess the relationship between the mechanical load elements and tillage depth according to the gear selection. The analysis of field test data showed that M2H showed a nonlinear trend in relatively shallow sections, while M3L and M3H showed only weak trends in the tillage depth. It is not appropriate to analyze nonlinear relationships using the Pearson correlation analysis method. Therefore, Pearson correlation analysis was performed on the deep tillage depth range (15–19 cm), which is significant from a tillage operation perspective. Pearson correlation coefficient results were classified into weak (±0.1–0.3), moderate (±0.3–0.5), and strong (> ±0.5) ranges. For M2H, analysis results showed a very weak Pearson correlation coefficient within the weak correlation level (±0.011–0.237). The front axle load, travel speed, and slip ratio at M2H were not related to the tillage depth. For M3L, engine load, rear axle rotational speed, travel speed, and fuel rate were moderately affected (±0.356–0.422) by the tillage depth. The front axle load and slip ratio, which were not affected by the tillage depth at M3L, also had a weak affect (±0.118–0.284) in M3L. For M3H, all mechanical elements, with the exception of the front axle torque (0.458), are shown to have strong relationships (±0.506–0.783). Overall, the front axle load was less affected by the tillage depth, and the engine load and fuel rate were more affected by the higher gear selection. The results of the Pearson analysis of correlation between the tillage depth and mechanical load elements are shown in Table 15.

## 4. Discussion

**Figure 11:**
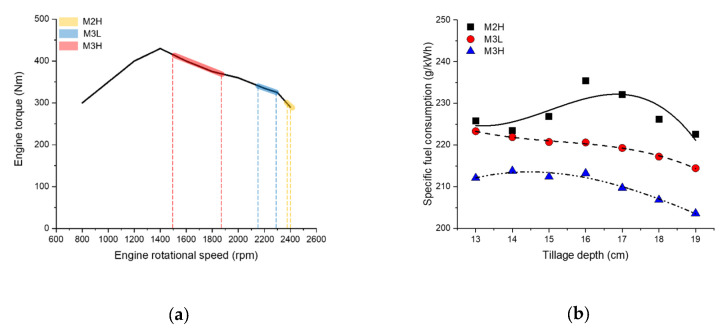
(**a**) Engine performance diagram of an agricultural tractor and (**b**) specific fuel consumption according to tillage depth and gear selections.

The analysis of the field test results shows that the higher the gear, the larger the average engine torque and the lower the average engine rotational speed. According to the engine performance diagram of the agricultural tractor used in this experiment, shown in Figure 11a [67], the maximum engine torque was 430 Nm at 1400 rpm, which is 132% of the rated torque. The overall average engine torque at M2H was 64.52% of the maximum and increased to 68.29% as the tillage depth increased. The average engine torque at M3L was 77.17% of the maximum, and as the tillage depth increased, the average engine torque increased by 79.49 Nm. In addition, the average engine speed at M3L was 2233.06 rpm, which was slower than M2H, but the fuel efficiency increased in terms of engine specifications. M3H had an average engine torque of 380.63 Nm, which was 88.51% of the maximum. In addition, the average engine torque at a tillage depth of 19 cm was 415.6 Nm, which is a high torque corresponding to 96.65% of the maximum.

For the specific fuel consumption as the fuel efficiency index considering the tractor output, low values correspond to a high fuel efficiency. The higher the gear selection and the deeper the tillage depth, the lower the specific fuel consumption. As the tillage depth became deeper, the engine and axle rpm were lowered due to the occurrence of high loads, resulting in low fuel consumption and increased fuel efficiency. The difference between the M2H with the lowest fuel efficiency and the M3H with the highest fuel efficiency among the gear stages corresponding to the recommended working speed range, is the difference in the overall average of 17.24 g/kWh (up to 22.39 g/kWh specific fuel efficiency difference), which shows an average fuel efficiency difference of 7.6% (up to 9.7% fuel efficiency difference). The results of the SFC according to tillage depth and gear selections is are shown in Figure 11b.

The overall average front and rear axle torques at the same tillage depth showed a higher value with higher gears for the deep tillage ranges (15–19 cm). The average front axle torque at M2H showed similar values without a significant effect for the tillage depth—much like the trend of engine torque. While tillage depth at M2H did not affect the slip ratio, the travel speed of M2H was reduced. This indicates that the axle rotational speed was reduced due to deteriorated engine rotational speed, but it is believed that the slip between the wheel and the ground rarely occurred. The M2H gear can work while overcoming the appropriate load at a level not significantly affected by the tillage depth, but it is not suitable in terms of saving time, as the overall average working speed is 0.37–0.5 km/h (6.7%–8.8%) slower than M3L and M3H. If the theoretical speed is not sufficient to secure the traction force, the influence of the mechanical load on the tillage depth is small, but the working efficiency is low because of the low travel speed. However, the theoretical speed of M3H is faster than M3L, but the slip ratio is 13.6% smaller. Nevertheless, the travel speed of M3H is 0.13 km/h (2.7%) slower than M3L. This means that the ground slip between the tire and the soil is small, but the reduction in the axle speed due to the lower engine speed has a significant effect on travel speed. For all tillage depth sections, the fuel efficiency of M3H is better than M3L. In addition, in the middle tillage depth section of 13–16 cm, it was possible to achieve a high working speed with high fuel efficiency, which is considered to be suitable for plow tillage in the middle tillage depth range. From the point of view of agricultural machinery design and the proper matching of tractors and implements, it is important to analyze axle loads through field tests [68,69,70]. Generally, the selection of gears that are suitable for farm operations should be considered in terms of fuel efficiency and working speed [71]. In addition, the maximum fuel efficiency and the minimum working time for the same tillage depth can be defined as the ideal farming operation parameters for the agricultural tractor. From this point of view, the M3H overcomes the high resistance torque produced by each major part at a high fuel efficiency and high working speed at the same tillage depth and has high traction performance during plow tillage. In summary, from the overall average point of view, the higher the average output, the higher the expected fuel rate consumption [72], but due to the high soil resistance at deep tillage depths, the high mechanical load caused a throttle down effect as reported in some previous studies [30,31]. According to the field results for this test, the higher the gear selection, the better the fuel efficiency. From the analysis, this effect was shown not to be due to the increase in engine power at the same fuel consumption but, rather, as similar to the throttle down due to a high mechanical load. Therefore, by excluding the gear stages in which the engine rpm hardly dropped due to the low mechanical load in the target tillage depth section (as in the case of M2H in this study), it is determined that higher fuel efficiency can be obtained for higher gear stages and deeper tillage depths.

## 5. Conclusions

In this study, the effect of tillage depth and gear selection on the mechanical load and fuel efficiency of an agricultural tractor (engine load, fuel rate, axle load, travel speed, and slip ratio) was analyzed during plow tillage. In field experiments, an agricultural field measuring system consisting of a tillage depth measurement part and a load measurement part was attached to an agricultural tractor to simultaneously measure cultivation depth and mechanical load elements. The measured data were analyzed at tillage depths of 13–19 cm estimated to correspond to the depth of hardpan, which is a significant section in terms of load analysis. The main results are as follows:

(1) From these results, plow tillage operation was shown to be performed at a greater tillage depth than the top estimated hardpan depth where instantaneous slopes are observed by cone penetration tests. While the total average tillage depth for each gear selection had a 0.17–1.14 cm difference, it was confirmed that the plow tillage in each stage was performed for a wide tillage depth section of 13–19 cm. Therefore, even if the average tillage depth is the same, soil–machine interactions may occur in various ways, depending on the gear selection, so studies that consider this should be conducted.
(2) The overall average travel speed at M2H was 5.23 km/h with an average slip ratio of 16.71%. For M3L, the overall average travel speed was 5.73 km/h with a highest average slip ratio of 19.13%. The overall average travel speed at M3H was 5.6 km/h with an average slip ratio of 16.54%. Despite the different theoretical speeds, the travel speed for M3L was found to be the fastest due to the reduction of axle rotational speed and the ground slip between the tire and the soil.
(3) Regarding the average engine torque, M3L and M3H tended to have lower engine rotational speeds and the engine torques rapidly increased with increasing tillage depths, and this was not case for the M2H gear. The front axle torque was the least affected by the tillage depth of the mechanical load elements. As the tillage depth increased, M3L and M3H tended to have higher engine loads, rear axle loads, and fuel efficiencies as the tillage depth increased. This seems to be affected by decreasing rotational speed, which was due to the high load that occurs when the tillage depth increases.
(4) While M3L has the highest working speed, M3H was chosen to overcome the high mechanical loads at high average driving speeds, with the highest fuel efficiency at the same tillage depth. It was confirmed that even for the three gear stages within the working speed range of the moldboard plow, the fuel efficiency differed by up to about 10% at the same tillage depth. It is judged that the most suitable gear for carrying out plow tillage in rice paddy field is the M3H. Therefore, in order to overcome the high load and obtain high fuel efficiency during the tillage operation, it is necessary to perform the plow tillage using M3H.

From these results, the analysis shows that tillage depth and gear selection greatly influenced the mechanical load and fuel efficiency of the agricultural tractor during plow tillage. Therefore, tillage depth and gear selection should be considered when analyzing the mechanical load of an agricultural tractor. To ensure reliable and effective tractor design, it is very important that the information about tillage depth and travel speed correspond to the gear used to perform the tillage operation. Although this study considered soil property, tillage depth and gear selection according to soil depth, it is considered that further follow-up studies are needed in various agricultural work environments with different types of operation.

In conclusion, the effect of tillage depth and gear selection on the mechanical load and fuel efficiency of the agricultural tractor during plow tillage was confirmed using a field measuring system. Considering the results of this study, it will be useful information to set field test conditions and to analyze the data of measured mechanical loads to be used toward the optimal design of agricultural tractors. In a future study, we plan to conduct a load analysis of agricultural machinery that considers the elements of soil mechanics and soil–machine interactions. In addition, the results of this study are considered to be useful for research on the design of tractor power transmission systems, DEM simulation model development, and fault diagnosis for the major components of agricultural machinery.

## Figures and Tables

**Table 1:** Specifications of the agricultural tractor.

Item		Specification
Weight	Empty (kg)	3985
	Total (kg)	5260
Length (mm) × width (mm) × height (mm)		4225 × 2140 × 2830
Engine	Rated power (kW)	78 @ 2300 rpm
	Max. torque (Nm)	430 @ 1400 rpm
Transmission	Main	4 stages and power shift (high, low)
	Sub	4 stages
Tire	Type	Bias
	Size	13.6–24 8PR, 18.4–34 10PR (front, rear)
	Pressure (psi)	31, 23 (front, rear)
	Lug height (mm)	36, 38 (front, rear)

**Table 2:** Specification of the attached implement used in this study.

Item	Specification
Type of implement	Moldboard plow
Weight (kg)	790
Length (mm) × width (mm) × height (mm)	2800 × 2150 × 1250
Required power (kW)	67–89
Maximum working depth (mm)	Up to 200
Travel speed (km/h)	5–8
Share type	Plain coulter with spring
Number of rows	8

**Table 3:** Mean soil property according to the depth of the soil.

Soil Property	Depth of Soil (cm)			
	0–5	5–10	10–15	15–20
Cone index (kPa)	558.12	1026.18	1219.74	2026.34
Soil water content (%)	30.3	38.2	37.6	33.8
Bulk Density ($\mathrm{kg}/m^{3}$)	1650	1800	1830	1900
Shear strength (kPa)	43.87	63.36	73.11	77.99

**Table 4:** Descriptive statistics of the measured tillage depth (cm) during plow tillage.

Gear Selection (Working Time, Sec)	Mean	Max.	Std.
M2H (49.6)	15.61	19.48	1.51
M3L (55.93)	16.58	18.9	1.47
M3H (60.07)	15.44	19.73	1.89

**Table 5:** Mean travel speed (km/h) according to tillage depth and gear selection (GS).

GS	Measured Tillage Depth (cm)							
	13	14	15	16	17	18	19	Total
M2H	5.1 ^Ab^	5.0 ^Aa^	5.21 ^Abc^	5.28 ^Ade^	5.34 ^Ae^	5.26 ^Acd^	5.11 ^Aa^	5.23
M3L	6 ^Cd^	5.84 ^Cc^	5.83 ^Cc^	5.83 ^Cc^	5.73 ^Cb^	5.51 ^Ca^	5.44 ^Ca^	5.73
M3H	5.9 ^Bf^	5.77 ^Be^	5.9 ^Bf^	5.63 ^Bd^	5.36 ^Bc^	5.03 ^Bb^	4.1 ^Ba^	5.6

^A,B,C,a,b,c,d,e,f^ Means within each row with the same combination of letters are not significantly different at *p* < 0.05 according to Duncan’s multiple range tests (capital letters: gear selection; lowercase letters: tillage depth).

**Table 6:** Mean slip ratio (%) according to tillage depth and gear selection (GS).

GS	Measured Tillage Depth (cm)							
	13	14	15	16	17	18	19	Total
M2H	17.23 ^Ac^	18.28 ^Ad^	16.76 ^Abc^	16.19 ^Aab^	15.39 ^Aa^	16.32 ^Abc^	18.3 ^Ad^	16.71
M3L	14.48 ^Ba^	16.93 ^Bb^	16.75 ^Bb^	16.42 ^Bb^	17.01 ^Bb^	18.66 ^Bc^	18.42 ^Bc^	17
M3H	13.94 ^Aa^	15.5 ^Ab^	15.1 ^Ab^	16.3 ^Ac^	17.75 ^Ad^	19.22 ^Ae^	25.57 ^Af^	16.54

^A,B,a,b,c,d,e,f^ Means within each row with the same combination of letters are not significantly different at *p* < 0.05 according to Duncan’s multiple range tests (capital letters: gear selection, lowercase letters: tillage depth).

**Table 7:** Mean engine torque (Nm) according to the tillage depth and gear selection (GS).

GS	Measured Tillage Depth (cm)							
	13	14	15	16	17	18	19	Total
M2H	282.28 ^Ac^	293.68 ^Ad^	281.83 ^Ac^	266.86 ^Aa^	266.67 ^Aa^	275.28 ^Ab^	283.6 ^Ac^	277.46
M3L	322.58 ^Ba^	328.29 ^Bb^	328.82 ^Bb^	329.52 ^Bb^	332.46 ^Bc^	336.92 ^Bd^	341.84 ^Be^	331.86
M3H	373.91 ^Ca^	376.38 ^Cb^	373.06 ^Ca^	380.06 ^Cc^	386.93 ^Cd^	394.45 ^Ce^	415.6 ^Cf^	380.63

^A,B,C,a,b,c,d,e,f^ Means within each row with the same combination of letters are not significantly different at *p* < 0.05 according to Duncan’s multiple range tests (capital letters: gear selection, lowercase letters: tillage depth).

**Table 8:** Mean engine rotational speed (rpm) according to tillage depth and gear selection (GS).

GS	Measured Tillage Depth (cm)							
	13	14	15	16	17	18	19	Total
M2H	2406.4 ^Cd^	2404.4 ^Cd^	2403.8 ^Ccd^	2402.7 ^Ccd^	2399.6 ^Cc^	2392.1 ^Cb^	2368.9 ^Ca^	2401.8
M3L	2290.4 ^Be^	2270.2 ^Bd^	2260.5 ^Bd^	2254.8 ^Bd^	2228.6 ^Bc^	2192.1 ^Bb^	2151.4 ^Ba^	2233
M3H	1877.5 ^Af^	1857.2 ^Ae^	1885.7 ^Af^	1826.2 ^Ad^	1766.8 ^Ac^	1691.5 ^Ab^	1497.3 ^Aa^	1818.8

^A,B,C,a,b,c,d,e,f^ Means within each row with the same combination of letters are not significantly different at *p* < 0.05 according to Duncan’s multiple range tests (capital letters: gear selection, lowercase letters: tillage depth).

**Table 9:** Mean engine power (kW) according to the tillage depth and gear selection (GS).

GS	Measured Tillage Depth (cm)							
	13	14	15	16	17	18	19	Total
M2H	71.09 ^Ac^	73.9 ^Ad^	70.91 ^Ac^	67.28 ^Aa^	66.98 ^Aa^	68.92 ^Ab^	70.31 ^Ac^	69.75
M3L	77.29 ^Cb^	78 ^Ce^	77.77 ^Cd^	77.73 ^Cd^	77.49 ^Cc^	77.25 ^Cb^	76.96 ^Ca^	77.5
M3H	73.46 ^Bf^	73.13 ^Be^	73.56 ^Bf^	72.38 ^Bd^	71.23 ^Bc^	69.56 ^Bb^	65 ^Ba^	72.19

^A,B,C,a,b,c,d,e,f^ Means within each row with the same combination of letters are not significantly different at *p* < 0.05 according to Duncan’s multiple range tests (capital letters: gear selection, lowercase letters: tillage depth).

**Table 10:** Mean fuel rate (L/h) according to the tillage depth and gear selection (GS).

GS	Measured Tillage Depth (cm)							
	13	14	15	16	17	18	19	Total
M2H	19.34 ^Bc^	19.89 ^Bd^	19.38 ^Bc^	19.08 ^Bb^	18.7 ^Ba^	18.78 ^Ba^	18.85 ^Ba^	19.24
M3L	20.79 ^Ce^	20.85 ^Ce^	20.68 ^Cd^	20.66 ^Cd^	20.47 ^Cc^	20.21 ^Cb^	19.88 ^Ca^	20.49
M3H	18.77 ^Ae^	18.83 ^Ae^	18.82 ^Ae^	18.59 ^Ad^	18 ^Ac^	17.33 ^Ab^	15.94 ^Aa^	18.4

^A,B,C,a,b,c,d,e^ Means within each row with the same combination of letters are not significantly different at *p* < 0.05 according to Duncan’s multiple range tests (capital letters: gear selection, lowercase letters: tillage depth).

**Table 11:** Mean front axle torque (Nm) according to the tillage depth and gear selection (GS).

GS	Measured Tillage Depth (cm)							
	13	14	15	16	17	18	19	Total
M2H	3807.5 ^Ad^	3841.8 ^Ad^	3729 ^Ac^	3621.1 ^Aab^	3561 ^Aa^	3657 ^Ab^	3927.4 ^Ae^	3697.7
M3L	3662.2 ^Ba^	3847.2 ^Bd^	3694 ^Bab^	3681 ^Ba^	3746.4 ^Babc^	3780 ^Bbcd^	3830.1 ^Bcd^	3728.1
M3H	3772.4 ^Ca^	3774 ^Ca^	3778 ^Ca^	3835.1 ^Cb^	3918.2 ^Cc^	4108.3 ^Cd^	4362.3 ^Ce^	3861.2

^A,B,C,a,b,c,d,e^ Means within each row with the same combination of letters are not significantly different at *p* < 0.05 according to Duncan’s multiple range tests (capital letters: gear selection, lowercase letters: tillage depth).

**Table 12:** Mean front axle rotational speed (rpm) according to the tillage depth and gear selection (GS).

GS	Measured Tillage Depth (cm)							
	13	14	15	16	17	18	19	Total
M2H	30.14 ^Ab^	29.93 ^Aa^	30.1 ^Ab^	30.34 ^Acd^	30.37 ^Ad^	30.26 ^Ac^	30.37 ^Ad^	30.19
M3L	34.01 ^Ce^	33.64 ^Cd^	33.54 ^Cd^	33.44 ^Ccd^	33.16 ^Cc^	32.62 ^Cb^	32.32 ^Ca^	33.19
M3H	32.77 ^Be^	32.79 ^Be^	33.43 ^Bf^	32.21 ^Bd^	31.36 ^Bc^	30.24 ^Bb^	26.81 ^Ba^	32.18

^A,B,C,a,b,c,d,e,f^ Means within each row with the same combination of letters are not significantly different at *p* < 0.05 according to Duncan’s multiple range tests (capital letters: gear selection, lowercase letters: tillage depth).

**Table 13:** Mean rear axle torque (Nm) according to the tillage depth and gear selection (GS).

GS	Measured Tillage Depth (cm)							
	13	14	15	16	17	18	19	Total
M2H	6887.3 ^Bc^	7222.8 ^Be^	6990.7 ^Bcd^	6618.4 ^Ba^	6654.5 ^Bab^	6761.3 ^Bb^	7032.6 ^Bd^	6857.2
M3L	6847.1 ^Ca^	6862.4 ^Ca^	6897 ^Cab^	6973.0 ^Cabc^	7039.7 ^Cbc^	7133.8 ^Cc^	7511.5 ^Cd^	7025.7
M3H	6688.3 ^Ac^	6615.4 ^Aab^	6573.3 ^Aa^	6655.3 ^Abc^	7013.4 ^Ad^	7199.8 ^Ae^	7607.3 ^Af^	6754.8

^A,B,C,a,b,c,d,e,f^ Means within each row with the same combination of letters are not significantly different at *p* < 0.05 according to Duncan’s multiple range tests (capital letters: gear selection, lowercase letters: tillage depth).

**Table 14:** Mean rear axle rotational speed (rpm) according to tillage depth and gear selection.

GS	Measured Tillage Depth (cm)							
	13	14	15	16	17	18	19	Total
M2H	21.41 ^Ab^	21.33 ^Aa^	21.47 ^Ac^	21.63 ^Ae^	21.63 ^Ae^	21.56 ^Ad^	21.44 ^Abc^	21.51
M3L	24.3 ^Cf^	24.1 ^Ce^	23.97 ^Cde^	23.88 ^Cd^	23.64 ^Cc^	23.23 ^Cb^	22.84 ^Ca^	23.67
M3H	23.5 ^Be^	23.42 ^Be^	23.8 ^Bf^	23.05 ^Bd^	22.28 ^Bc^	21.35 ^Bb^	18.87 ^Ba^	22.94

^A,B,C,a,b,c,d,e,f^ Means within each row with the same combination of letters are not significantly different at *p* < 0.05 according to Duncan’s multiple range tests (capital letters: gear selection, lowercase letters: tillage depth).

**Table 15:** Pearson correlation analysis results between tillage depth and mechanical load elements.

VAR	$T_{engine}$	$RPM_{engine}$	$T_{front}$	$RPM_{front}$	$T_{rear}$	$RPM_{rear}$	TS	SR	FC
${\mathrm{TD}}_{M2H}$	0.139 *	−0.175 *	0.060 *	0.031 *	0.118 *	−0.124 *	−0.037	0.011	−0.237 *
${\mathrm{TD}}_{M3L}$	0.356 *	−0.394 *	0.118 *	−0.284 *	0.120 *	−0.370 *	−0.360 *	0.232 *	−0.422 *
${\mathrm{TD}}_{M3H}$	0.575 *	−0.584 *	0.458 *	−0.531 *	0.506 *	−0.584 *	−0.657 *	0.567 *	−0.783 *

* Significant difference at *p* < 0.05. (TD = tillage depth; T_engine_ = engine torque; RPM_engine_ = engine rotational speed; T_front_ = front axle torque; RPM_front_ = front axle rotational speed; T_rear_ = rear axle torque; RPM_rear_ = rear axle rotational speed; TS = travel speed; SR = slip ratio; FC = fuel rate).
